# Dental patient reported outcome and oral health-related quality of life measures: protocol for a systematic evidence map of reviews

**DOI:** 10.1038/s41405-021-00065-6

**Published:** 2021-01-28

**Authors:** Darragh Beecher, Patrice James, John Browne, Zelda Di Blasi, Máiréad Harding, Helen Whelton

**Affiliations:** 1 Oral Health Services Research Centre, University College Cork, Wilton, Cork, T12 E8YV Ireland; 2 School of Applied Psychology, University College Cork, Cork, T23 TK30 Ireland; 3 School of Public Health, University College Cork, Cork, T12 XF62 Ireland; 4 Cork University Dental School & Hospital, University College Cork, Wilton, Cork, T12 E8YV Ireland; 5 Cork Kerry Community Healthcare Area, Health Services Executive, Dental Clinic, St. Finbarr’s Hospital, Cork, T12 XH60 Ireland; 6 College of Medicine & Health, University College Cork, Cork, T12 EKD0 Ireland

**Keywords:** Oral-health-related quality of life, Dental epidemiology

## Abstract

**Aims:**

This research synthesis protocol addresses the question: what is the evidence concerning measurement properties of dental patient reported outcome measures (dPROMs), and regarding the real-world value of dPROMs, and where are the gaps in this evidence? Evidence mapping will systematically examine reviews of quantitative dPROMs used to assess the impact of oral health on the quality of life of dental patients and research participants. Evidence gaps where future research or systematic reviews are required will be identified.

**Materials and methods:**

This protocol accords with the PRISMA-P guideline. Open Science Framework Registration 10.17605/OSF.IO/RZD3N. Biomedical and grey literature databases will be searched, adapting the same search strategy. Published or unpublished reviews evaluating any dPROM will be considered for inclusion. There will be no restriction by date, setting, or language. AMSTAR2 and ROBIS will evaluate risk of bias. Psychometric criteria will be adapted from COSMIN. Data will be summarised separately for specific populations and conditions.

**Discussion:**

The findings will enable clinicians and researchers to identify methodologically robust dPROMs, appropriate for use with relevant populations and conditions. Implications for real-world practice and research will be discussed.

## Introduction

Oral health is a subjective, dynamic, multidimensional construct, encompassing physical, psychological, social, and emotional domains, and is integral to overall health and wellbeing. Good oral health enables eating, speaking, smiling, and socialising, without experiencing embarrassment, discomfort, or pain.^1^ Across the life course, the chronic, progressive nature of oral conditions has substantial negative impacts on individuals. Yet, oral diseases remain highly prevalent, affecting 3.5 billion people worldwide.^1^ Patient reported outcomes (PROs) capture patients’ perceptions of the impact of disease or treatment (or both) on symptoms, functioning, and health-related quality of life. A dental patient reported outcome (dPRO) is a narrower construct, relating specifically to patients’ subjective experience of their oral health, and has been defined as any report of the status of a patient’s oral health condition that comes directly from the dental patient, without interpretation of their response by a clinician or anyone else.^2,3^ dPROs are measured by dental patient reported outcome measures (dPROMs), typically multi-item questionnaires completed by patients. Proxy reporting is confined to parents of young children, ideally reporting only those events or behaviours that can be observed.^2^ Inclusion of the public’s evaluation of the impact of their oral health on their lives is consistent with best-practice in dental research and evidence-based dentistry,^4,5^ and provides additional evidence regarding the impact of oral health, dental conditions, and their treatment on outcomes that matter most to patients.^6–8^

Oral health-related quality of life (OHRQoL) is the most important dPRO. More than 50 OHRQoL measures have been developed for use with paediatric and adolescent, adult, and older dental populations.^9–11^ The relationship between oral health, dental conditions and diseases, and OHRQoL, has been explored theoretically^12^ and through diverse research and reviews.^13^ There is emerging consensus among recommendations that advocate for the inclusion of dPROs and OHRQoL in dentistry^14,15^ and dPRO developers^16^ that OHRQoL has a four-dimensional structure. Together, they suggest that orofacial symptoms and pain, oral function and limitations, psychosocial impacts, and orofacial aesthetics are valuable dPRO concepts, alongside OHRQoL.

To inform the development of this protocol, we conducted a preliminary search based on Sischo and Broder’s^17^ highly cited literature review. They reported 300 OHRQoL articles published in the 20-year period 1990–2010, with 214 of these published 2006–10. Repeating their published search strategy for PubMed and ProQuest almost 10 years later, on August 13th 2020, confirmed continued growth in publishing on this topic. Table 1 shows that 305 OHRQoL papers were published 2011–2015, with 504 more 2016–2020. The authors noted the relatively small number of OHRQoL review articles published up to 2010, identifying a need for systematic reviews of the literature. Perhaps heeding this, two-thirds of all OHRQoL reviews now retrieved by their search strategy are published since the beginning of 2016. Within these search results, no overviews were identified, including no scoping reviews nor evidence maps.

**Table 1:** Timeline of OHRQoL research across disciplines in PubMed and ProQuest*.

Discipline Search strategy	*1990–1995*	*1996–2000*	*2001–2005*	*2006–2010 (Nov)*	2011 (Dec 2010)–2015	2016–2020 (Aug)	Reviews		
							*1990–2010*	2011–2015	2016–2020
Pediatric (OHRQoL) AND (children)	*0*	*2*	*19*	*70*	130	225	*7*	1	15
Orthodontic (OHRQoL) AND (orthodontic)	*0*	*1*	*9*	*35*	41	80	*1*	3	7
Prosthodontic (OHRQoL) AND (prosthodontic)	*0*	*0*	*4*	*24*	17	16	*3*	0	3
Geriatric (GOHAI OR (OHIP AND elderly OR old))	*2*	*6*	*25*	*57*	76	79	*0*	0	3
Oral medicine / surgery (OHRQoL) AND (oral surgery OR oral cancer)	*2*	*1*	*9*	*20*	20	57	*2*	0	5
Craniofacial (OHRQoL) AND (craniofacial OR orofacial OR cleft)	*0*	*0*	*6*	*8*	21	47	*2*	2	6
Total	***4***	***10***	***72***	***214***	**305**	**504**	***15*** **25%**	**6** **10%**	**39** **65%**

*Data 1990–2010 from Appendix Table 1 & 2.^17^

To avoid ethical pitfalls and waste of resources, clinicians and researchers must select dPROMs that are valid and reliable, responsive, interpretable, and feasible in relation to their research question and the relevant population(s), condition(s), and context(s).^18,19^ From the rapid search described above, we identified 45 reviews and more than 800 studies concerning OHRQoL published during the last decade. With such abundance of prior research, clinicians and researchers may struggle to identify the most applicable dPROM due to discrepant findings among the numerous studies and reviews of measurement properties and condition-specific OHRQoL reviews available.^20^ Further, use of heterogenous dPROMs, even when appropriately selected, limits the extent to which findings can be interpreted and compared to other studies, and restricts their inclusion in research syntheses, such as meta-analysis, weakening the evidence base. Core outcome sets could potentially address such issues, but to date few have been developed in dentistry.^8^ A comprehensive synthesis of the current state of the evidence concerning dPROMs appears timely.

Evaluation of the need for new or updated systematic reviews using the Panel for Updating Guidance for systematic reviews (PUGs) Checklist,^21^ and consideration of guidance on choosing a review approach,^22,23^ concluded with the identification of evidence mapping as an appropriate methodology for this review.^24^ An evidence map is a systematic search of a broad field to identify gaps in knowledge and/or future research needs that presents results in a user-friendly format, often a visual figure or graph, or a searchable database.^25^ Evidence maps identify what evidence there is, and where gaps lie, but do not synthesise what the evidence says as per a systematic review.^26^

## Aims

Our research questions are:

1. What is the evidence concerning measurement properties of dPROMs?
2. What is the evidence regarding the real-world value of dPROMs; for example, responsiveness to treatment benefits and ability to discriminate between different patient groups?
3. Where are the gaps in the evidence?

## Methods and materials

This protocol is reported per the Preferred Reporting Items for Systematic review and Meta-Analysis for Abstracts (PRISMA-A)^27^ and Protocols (PRISMA-P)^28^ checklists, adapted for evidence mapping, and reporting of the main review will accord with PRISMA-ScR, for Scoping Reviews.^29^ There are many similarities between evidence map and scoping review methods, so in the absence of a guideline for conducting evidence mapping, our review methods are adapted from the *JBI Manual for Evidence Synthesis—Scoping Reviews*.^30^ The guidance on scoping reviews in the recent *JBI* update and PRISMA-ScR are mutually consistent.^29,30^ For clarity, we refer to evidence mapping throughout, though individual references may originally pertain to scoping review methods. This protocol was prospectively registered with the Open Science Framework on September 29th, 2020 (Registration 10.17605/OSF.IO/RZD3N). In the event of protocol amendments, the details of each change will be tabulated; changes will not be incorporated into the protocol.^28^

### Inclusion criteria

#### Types of evidence sources

This evidence mapping will be conducted at a level similar to a “review of reviews” approach. Published or unpublished systematic and non-systematic reviews considering any dPROM will be considered for inclusion as older reviews tend not to have used formal systematic methods. The findings of this high-level review will indicate if a more extensive research synthesis which includes primary studies is warranted. Editorials and opinion pieces will be excluded, as will reviews of outcome measures of patient satisfaction or experience.

For inclusion, each review must evaluate one or more dPROMs regarding either measurement properties or their real-world value (e.g., responsiveness to treatment benefits, or ability to discriminate between different patient groups). Reviews of all quantitative dPROMs for oral health are of interest, including condition-specific, functional, and symptom-based measures. Generic physical and/or mental health PROMs are beyond the dental focus of our review. Reviews incorporating proxy-reported measures completed by parent/caregiver will be considered for inclusion where children cannot provide responses independently due to their young age.

#### Population, concept, and context

The core concepts included will be OHRQoL, orofacial symptoms and pain, oral function and limitations, psychosocial impacts and orofacial aesthetics. The population and context of interest are paediatric and adolescent, adult, and older dental patients and research participants undergoing any dental treatment, including screening.

No date restriction will be applied. There will be no restriction by language, but as the search will be conducted through English, the existence of an English title and abstract are required to assess the relevance of the study. Details of non-English language reviews will be tabulated but excluded from the review. Translation and cross-cultural adaptation is an important aspect of dPROM development and selection in its own right,^31^ and is beyond the scope of this review.

### Search strategy

The search strategy balances recommendations for high sensitivity^32^ against feasibility,^33,34^ and was guided by literature regarding searching in systematic reviews^35^ as well as in overviews of systematic reviews.^36^

Databases will be searched from inception. MEDLINE via PubMed and Epistemonikos will be searched, complemented by reference checking of included studies, as this has been identified as the best database combination to identify systematic reviews of health-related topics.^37^ Embase, Web of Science, and Google Scholar will also be searched as a minimum requirement to guarantee adequate and efficient coverage in systematic reviews. The COSMIN database and PsycINFO will be included as our review topic is related to the focus of these databases.^38^ TRIP will be searched to facilitate the identification of reviews at the protocol stage, and to explore comparison with their automated evidence mapping system, if possible. Citation searches for the first publication and validation study associated with each dPROM included in one or more reviews will be conducted to identify any additional reviews that have also included the instrument.^39^

The search strategy will identify relevant dPROM reviews based on the core concepts or constructs, and publication type. The population and context will not be specified in the search as dPROMs for all age groups used in either clinical or research settings are relevant to the review.

As our evidence map concerns systematic and non-systematic reviews, we will include a search for the required publication types. We will use the most sensitive version of an optimal search strategy for retrieving systematic reviews from MEDLINE to ensure all relevant reviews are included in the evidence map.^40^ This strategy will be supplemented by the more recently developed PubMed systematic reviews filter which encompasses citations assigned the “Systematic Review” publication type during MEDLINE indexing, citations that have not yet completed MEDLINE indexing, and non-MEDLINE citations.^41^ The full PubMed search strategy is presented in Table 2.

**Table 2:** PubMed search strategy.

Aspect	Search query	
Constructs	OHRQoL	#1. (Oral health[MeSH] AND quality of life[MeSH]) OR (Oral Health[tiab] AND Quality of Life[tiab]) OR oral health-related quality of life[tiab] OR OHRQoL[tiab] OR ((oral health[MeSH] OR (oral[tiab] AND health[tiab]) OR oral health[tiab]) AND related[tiab] AND (quality of life[MeSH] OR (quality[tiab] AND life[tiab]) OR quality of life[tiab]))
		#2. Sociodental[tiab]
		#3. #1 OR #2
	QoL	#4. Quality of life[MeSH] OR (quality[tiab] AND life[tiab]) OR quality of life[tiab]
	Orofacial symptoms and pain	#5. Facial pain[MeSH] OR (facial[tiab] AND pain[tiab]) OR facial pain[tiab] OR (orofacial[tiab] AND pain[tiab]) OR orofacial pain[tiab]
	Oral function and limitations	#6. Oral function[tiab]
	Psychosocial impact	#7. Psychosocial impact[tiab] OR psychological wellbeing[tiab] OR psychological well-being[tiab] OR emotional wellbeing[tiab] OR emotional well-being[tiab] OR social wellbeing[tiab] OR social well-being[tiab] OR dental anxiety[MeSH] OR (dental[tiab] AND anxiety[tiab]) OR dental anxiety[tiab]
	Orofacial aesthetics	#8. Orofacial appearance[tiab] OR orofacial aesthetics[tiab] OR dental aesthetics[MeSH]
		#9. #4 OR #5 OR #6 OR #7 OR #8
	Dental speciality	#10. Dental public health[tiab] OR endodontic[tiab] OR restorative[tiab] OR oral medicine[tiab] OR oral surgery[tiab] OR orthodontic[tiab] OR periodontic[tiab] OR prosthodontic[tiab] OR paediatric dentistry[tiab] OR special care dentistry[tiab] OR ((geriatric[tiab] OR older[tiab]) AND (dental[tiab] OR dentistry[tiab]))
		#11. #9 AND #10
		#12. #3 OR #11
Publication Type		#13. search*[tiab] OR meta-analysis[pt] OR meta-analysis[tiab] OR meta-analysis[MeSH] OR review[pt] OR diagnosis[sh] OR associated[tiab]
		#14. (((systematic review[ti] OR systematic literature review[ti] OR systematic scoping review[ti] OR systematic narrative review[ti] OR systematic qualitative review[ti] OR systematic evidence review[ti] OR systematic quantitative review[ti] OR systematic meta-review[ti] OR systematic critical review[ti] OR systematic mixed studies review[ti] OR systematic mapping review[ti] OR systematic cochrane review[ti] OR systematic search and review[ti] OR systematic integrative review[ti]) NOT comment[pt] NOT (protocol[ti] OR protocols[ti])) NOT MEDLINE[subset]) OR (Cochrane Database Syst Rev[ta] AND review[pt]) OR systematic review[pt]
		#15. #13 OR #14
#16. #12 AND #15		

^*^The wildcard symbol. This means that search*[tiab] will retrieve results that include searched[tiab], searching[tiab], searches[tiab], etc.

This PubMed search strategy will be adapted for use with other databases. During the conduct of the search, familiarity with the evidence base will increase. Any useful search terms or sources identified will be incorporated. The final search strategy will be published in full, with amendments clearly identifiable.^30^

Grey literature, such as internal reports and working papers will be included in our search to reduce the impact of publication bias.^42,43^ We will search grey literature databases, including OpenGrey.^44^ However, strategies such as contacting key informants or third sector organisations will not be used as this would reduce the replicability of our search due to variability in responses.^42^

### Source of evidence selection

All records retrieved by the search strategy will be imported and managed using Endnote X9, with additional information stored in Microsoft Excel for Microsoft 365. A data charting form will be developed to facilitate data extraction from titles and abstracts. After de-duplication, a calibration exercise will take place in which three reviewers (D.B., P.J., Z.D.B.) will independently screen 20 randomly selected titles and abstracts against the eligibility criteria using the form. Discrepancies between reviewers will be discussed; modifications to the form and further calibration will take place as required. Once near perfect agreement (>80%) is achieved, screening will continue with one reviewer (D.B.) and one verifier (P.J. or Z.D.B.).

Potentially relevant full-text articles will be retrieved. Studies considered eligible by only one reviewer will be discussed; where consensus regarding inclusion or exclusion cannot be achieved, the third reviewer will make a final decision. Reasons for excluding full-text studies will be recorded. A PRISMA flow chart will be completed to illustrate studies included and excluded at each stage of the study selection process.^29^

### Data extraction

A data charting form will be developed to facilitate data extraction from each full-text review. Piloting will initially be conducted using two randomly selected reviews and the charting form modified as required before full data extraction begins. A further review will take place after data extraction from the first five studies to review the form and ensure its consistent use. Once near perfect agreement (>80%) is achieved, data extraction will continue with one reviewer (D.B.) and one verifier (P.J. or Z.D.B.), with any discrepancies resolved through discussion and the involvement of the third reviewer, if necessary.

Data extraction will include the following domains^30^:

- Study publication details
- Status of study: completed or ongoing
- Geographical coverage, if applicable
- Aims/purpose
- Review methods (e.g. systematic or non-systematic review; use of COSMIN, EMPRO or other criteria,^45^ if applicable)
- Inclusion criteria of systematic review
  - Population(s), including condition(s)
  - Concept(s)
  - Context(s)
  - Names of dPROMs
  - Measurement properties
- Critical appraisal approach to included studies, if applicable
- Outcomes regarding measurement properties of dPROMs or real-world value of dPROMs, including method of measurement
- Key findings as they relate to our mapping review questions

### Analysis of the evidence

Data from reviews of dPROMs will be categorised and summarised separately for paediatric and adolescent, adult, and older populations, and for different oral conditions. A summary of literature characteristics will be depicted in tabular format, including countries and contexts in which studies were conducted, review methods, inclusion criteria, number and name of dPROMs reviewed, characteristics of the study populations and oral conditions, and outcomes related to the real-world value of dPROMs.^30^

Based on a recently published meta-review of tools to assess the measurement properties of QoL instruments,^45^ we will use the comprehensive COSMIN criteria to structure a cross-tabular summary of measurement properties reported in included reviews. We will additionally summarise reporting of feasibility, including burden and fairness, and use of measures for non-evaluative purposes, areas where COSMIN is less well developed.^45^

As there is no specific guideline for the evaluation of the methodological quality of reviews of PROMs,^46^ critical appraisal of the quality of included dPROM reviews will be conducted using A MeaSurement Tool to Assess systematic Reviews 2 (AMSTAR2)^47^ adapted for PROM reviews,^48^ and the Risk of Bias in Systematic Reviews (ROBIS) tool.^49^ Though critical appraisal of individual sources of evidence is considered an optional aspect of evidence mapping,^29^ the methodological quality of knowledge synthesis in reviews of dPROMs is relevant to this review as differences in methodology may account for discrepant findings between included studies.

Older guidance recommends thematic analysis as part of the process of data analysis.^30^ However, there is a paucity of methodological transparency around how this might be conducted.^50^ We will consider the feasibility and appropriateness of this recommendation in the context of our aims, data extracted from included studies, and current methodological guidance.^30^ We will document our decision and reasons for it in our report.

### Presentation of the results

Tabular summaries will be adapted to the characteristics of the included reviews to capture information relevant to our aims, including recording of excluded reviews and records of quality assessment. Specifically, the number and publication trends of dPROM reviews concerning measurement properties and their real-world value will be identified; reviews related to specific populations and conditions will be noted. To estimate the likelihood of new primary research articles being omitted due to our focus on review articles, we will report the number of studies published since the search date of the most recent review article concerning each population or condition, though we will not review these primary studies. This will provide an estimate of how much new literature has been published since the period covered by the included reviews.

Conclusions regarding the quality of dPROMS will be summarised from reviews of measurement properties. High quality dPROMs will be identified with respect to specific populations and conditions. Evidence concerning the real-world value of dPROMS in terms of responsiveness to treatment benefit and ability to discriminate between different patient groups will be described. The feasibility of comparisons between the impact of different oral conditions on OHRQoL will be considered. Evidence gaps will be identified, including populations, conditions, or constructs that are not adequately assessed by existing dPROMs, or where further research or systematic reviews including primary studies are required. A descriptive summary will accompany the tabulated results.^30^

The format of the visual representation (i.e., the evidence map) will be selected to facilitate access and usability of the findings by clinicians and researchers seeking to include high quality dPROMs in dental surveys and studies of dental interventions or treatment,^24,26^ and those seeking to conduct research or a systematic review to further the evidence concerning dPROMs. The appropriateness of options such as bubble plots, 2D-and 3D-matrices, a matrix of evidence, and use of pivot tables and charts will be considered. The visual representation will also be accompanied by a descriptive report.^30^ We will publish the review in a peer-reviewed journal.

## Discussion

This systematic evidence map of reviews will enable clinicians and dental researchers to identify methodologically robust dPROMs, appropriate for use with specific populations and oral conditions. Gaps in the evidence base will be highlighted to inform future research, including identification of the need for a research synthesis which includes primary studies. Implications for real-world research, practice and policy will be discussed.

## References

[1] Peres MA (2019). Oral diseases: a global public health challenge. Lancet.

[2] U.S. Food and Drug Administration. Guidance for Industry Patient-Reported Outcome Measures: Use in Medical Product Development to Support Labeling Claims. (Department of Health and Human Services, Food and Drug Administration; Center for Drug Evaluation and Research (CDER), Center for Biologics Evaluation and Research (CBER), Center for Devices and Radiological Health (CDRH), Rockville, Maryland: U.S., 2009).

[3] John MT (2018). Health Outcomes Reported by Dental Patients. J. Evid. Based Dent. Pract..

[4] Sarkis-Onofre R (2015). Use of guidelines to improve the quality and transparency of reporting oral health research. J. Dent..

[5] Reissmann DR (2019). Dental patient-reported outcome measures are essential for evidence-based prosthetic dentistry. J. Evid. Based Dent. Pract..

[6] Whitlock EP (2010). AHRQ series paper 3: identifying, selecting, and refining topics for comparative effectiveness systematic reviews: AHRQ and the effective health-care program. J. Clin. Epidemiol..

[7] Hua F (2019). Increasing the value of orthodontic research through the use of dental patient-reported outcomes. J. Evid. Based Dent. Pract..

[8] Fleming PS, Koletsi D, O’Brien K, Tsichlaki A, Pandis N (2016). Are dental researchers asking patient-important questions? A scoping review. J. Dent..

[9] Zaror C (2019). Assessing oral health-related quality of life in children and adolescents: a systematic review and standardized comparison of available instruments. Clin. oral. Investig..

[10] Mittal H, John MT, Sekulic S, Theis-Mahon N, Rener-Sitar K (2019). Patient-reported outcome measures for adult dental patients: a systematic review. J. Evid. Based Dent. Pract..

[11] Hebling E, Pereira AC (2007). Oral health-related quality of life: a critical appraisal of assessment tools used in elderly people. Gerodontology.

[12] Sekulic S, Theis-Mahon N, Rener-Sitar K (2019). A systematic scoping review of oral health models. Qual. Life Res..

[13] Sekulic S, John MT, Haggman-Henrikson B, Theis-Mahon N (2020). Dental patients’ functional, pain-related, aesthetic, and psychosocial impact of oral conditions on quality of life—Project overview, data collection, quality assessment, and publication bias. J Oral Rehabil.

[14] FDI World Dental Federation. (2016). FDI policy statement on oral health and quality of life: Adopted by the FDI General Assembly: 24 September 2015, Bangkok, Thailand. Int. Dent. J..

[15] Pitts NB, Carter NL, Tsakos G (2018). The Brussels statement on the future needs for caries epidemiology and surveillance in Europe. Community Dent. Health.

[16] John MT (2020). Foundations of oral health-related quality of life. J Oral Rehabil.

[17] Sischo L, Broder HL (2011). Oral health-related quality of life: what, why, how, and future implications. J. Dent. Res..

[18] Ioannidis JP (2014). Increasing value and reducing waste in research design, conduct, and analysis. Lancet.

[19] Calvert M (2013). Reporting of patient-reported outcomes in randomized trials: the CONSORT PRO extension. JAMA.

[20] Richards D (2018). Too many reviews too few trials. Evid. Based Dent..

[21] Garner P (2016). When and how to update systematic reviews: consensus and checklist. BMJ.

[22] Munn Z (2018). Systematic review or scoping review? Guidance for authors when choosing between a systematic or scoping review approach. BMC Med Res Methodol..

[23] Grant MJ, Booth A (2009). A typology of reviews: an analysis of 14 review types and associated methodologies. Health Info Libr J..

[24] Snilstveit B, Vojtkova M, Bhavsar A, Stevenson J, Gaarder M (2016). Evidence & Gap Maps: A tool for promoting evidence informed policy and strategic research agendas. J. Clin. Epidemiol..

[25] Miake-Lye IM, Hempel S, Shanman R, Shekelle PG (2016). What is an evidence map? A systematic review of published evidence maps and their definitions, methods, and products. Syst. Rev..

[26] Saran A., White H. Evidence and gap maps: a comparison of different approaches (The Campbell Collaboration, Oslo, Norway, 2018). 10.4073/cmdp.2018.2 PMC8428058 37131398

[27] Beller EM (2013). PRISMA for Abstracts: reporting systematic reviews in journal and conference abstracts. PLoS Med..

[28] Shamseer L (2015). Preferred reporting items for systematic review and meta-analysis protocols (PRISMA-P) 2015: elaboration and explanation. BMJ.

[29] Tricco AC (2018). PRISMA Extension for Scoping Reviews (PRISMA-ScR): Checklist and Explanation. Ann. Intern Med..

[30] Peters M. D. J., *et al* Chapter 11: Scoping reviews. in *JBI Manual for Evidence Synthesis*. (eds. Aromataris E., Munn Z) (JBI, Adelaide, Australia, 2020)

[31] Wild D (2005). Principles of good practice for the translation and cultural adaptation process for patient-reported outcomes (PRO) measures: report of the ISPOR task force for translation and cultural adaptation. Value Health.

[32] Centre for Reviews and Dissemination. Systematic reviews: CRD’s guidance for undertaking reviews in health care. (University of York, York, UK, 2008).

[33] Buchberger B., Krabbe L., Lux B., Mattivi J. T. Evidence mapping for decision making: feasibility versus accuracy—when to abandon high sensitivity in electronic searches. *Ger Med Sci*. 2016;14:Doc09. 10.3205/000236 PMC4951635 27499726

[34] Parkhill AF (2011). Searches for evidence mapping: effective, shorter, cheaper. J. Med Libr Assoc..

[35] Cooper C., Booth A., Varley-Campbell J., Britten N., Garside R. Defining the process to literature searching in systematic reviews: a literature review of guidance and supporting studies. *BMC Med Res Methodol.* **18**, 1–14 (2018). 10.1186/s12874-018-0545-3 PMC6092796 30107788

[36] Lunny C., Brennan S. E., McDonald S., McKenzie J. E. Toward a comprehensive evidence map of overview of systematic review methods: paper 1—purpose, eligibility, search and data extraction. *Syst Rev*. **6**, 1–27 (2017). 10.1186/s13643-017-0617-1 PMC5698938 29162130

[37] Goossen K, Hess S, Lunny C, Pieper D (2020). Database combinations to retrieve systematic reviews in overviews of reviews: a methodological study. BMC Med Res Methodol..

[38] Bramer WM, Rethlefsen ML, Kleijnen J, Franco OH (2017). Optimal database combinations for literature searches in systematic reviews: a prospective exploratory study. Syst. Rev..

[39] Linder SK, Kamath GR, Pratt GF, Saraykar SS, Volk RJ (2015). Citation searches are more sensitive than keyword searches to identify studies using specific measurement instruments. J. Clin. Epidemiol..

[40] Montori VM, Wilczynski NL, Morgan D, Haynes RB, Hedges T (2005). Optimal search strategies for retrieving systematic reviews from Medline: analytical survey. BMJ.

[41] U.S. National Library of Medicine. Search Strategy Used to Create the PubMed Systematic Reviews Filter Bethesda, Maryland: National Library of Medicine. 2018. https://www.nlm.nih.gov/bsd/pubmed_subsets/sysreviews_strategy.html.

[42] Adams J (2016). Searching and synthesising ‘grey literature’ and ‘grey information’ in public health: critical reflections on three case studies. Syst. Rev..

[43] Paez A (2017). Grey literature: an important resource in systematic reviews. J Evid Based Med.

[44] Canadian Agency for Drugs and Technologies in Health. Grey Matters: a practical tool for searching health-related grey literature. (Canadian Agency for Drugs and Technologies in Health, Ottawa, Canada, 2018).

[45] Lorente S, Viladrich C, Vives J, Losilla JM (2020). Tools to assess the measurement properties of quality of life instruments: a meta-review. BMJ Open..

[46] Ma LL (2020). Methodological quality (risk of bias) assessment tools for primary and secondary medical studies: what are they and which is better?. Mil. Med Res..

[47] Shea BJ (2017). AMSTAR 2: a critical appraisal tool for systematic reviews that include randomised or non-randomised studies of healthcare interventions, or both. BMJ.

[48] Terwee CB (2016). The quality of systematic reviews of health-related outcome measurement instruments. Qual. Life Res..

[49] Whiting P (2016). ROBIS: A new tool to assess risk of bias in systematic reviews was developed. J. Clin. Epidemiol..

[50] Campbell M, Katikireddi SV, Sowden A, Thomson H (2019). Lack of transparency in reporting narrative synthesis of quantitative data: a methodological assessment of systematic reviews. J. Clin. Epidemiol..

